# Partial PFO Closure for Persistent Hypoxemia in a Patient with Ebstein Anomaly

**DOI:** 10.1155/2015/531382

**Published:** 2015-04-07

**Authors:** S. A. Zuberi, S. Liu, J. W. Tam, F. Hussain, D. Maguire, M. Kass

**Affiliations:** ^1^Department of Internal Medicine, Faculty of Medicine, University of Manitoba, 409 Tache Avenue, Winnipeg, MB, Canada R2H 2A6; ^2^Section of Cardiology, Department of Internal Medicine, Faculty of Medicine, University of Manitoba, 409 Tache Avenue, Winnipeg, MB, Canada R2H 2A6; ^3^Department of Anesthesia and Preoperative Medicine, Faculty of Medicine, University of Manitoba, 409 Tache Avenue, Winnipeg, MB, Canada R2H 2A6

## Abstract

Ebstein anomaly is characterized by deformities of the anterior leaflet of the tricuspid valve and atrialization of the right ventricle. Patients with severe tricuspid regurgitation are recommended to have tricuspid valve surgery with concomitant atrial septal defect closure. A 73-year-old female with Ebstein anomaly presented with severe hypoxemia. Transthoracic echocardiography revealed severe tricuspid regurgitation and a patent foramen ovale with right-to-left shunting. Complete percutaneous patent foramen ovale closure led to acute decompensation; however, partial closure led to hemodynamic stability and improved oxygenation. In conclusion, similar patients with “patent foramen ovale dependency” from longstanding shunts may benefit from partial patent foramen ovale closure.

## 1. Introduction

Ebstein anomaly, first described in 1866 by Dr. Wilhelm Ebstein, accounts for less than 1% of congenital heart defects [1]. It is characterized by deformities of the anterior leaflet of the tricuspid valve and displacement of septal and posterior leaflets into the right ventricle [1]. This leads to atrialization of the right ventricle with consequential small functioning ventricle [1]. More than 50% of patients with Ebstein anomaly have an atrial septal defect or patent foramen ovale, which may cause right-to-left shunting [2].

Ebstein anomaly patients with severe tricuspid regurgitation and atrial septal defects are recommended to have tricuspid valve surgery with simultaneous atrial septal defect closure [2]. Indications for surgery in adult Ebstein anomaly patients otherwise include progressively worsening exercise capacity, symptoms consistent with New York Heart Association classes III-IV, failure to thrive, progressive cardiomegaly, evidence of cyanosis, progressively worsening left or right ventricular function, and overwhelming tachyarrhythmias [2].

We present a patient with Ebstein anomaly unable to tolerate complete closure of a patent foramen ovale. To our knowledge, similar case has not been described in the adult population.

## 2. Case Report

Our patient was a 73-year-old female with history of Ebstein anomaly, Wolff-Parkinson-White syndrome, atrial fibrillation, hypertension, and chronic obstructive pulmonary disease on home oxygen, presented to the hospital with 1-week history of progressive shortness of breath on exertion and peripheral edema. Transthoracic echocardiography performed 2 years ago revealed mild tricuspid regurgitation and a suspected patent foramen ovale with left-to-right shunt by color Doppler.

On presentation, she had severe hypoxemia with room air oxygen saturation of 66% and partial pressure oxygen of 33 mmHg. A trial of bilevel positive airway pressure led to no improvement. She was started on empiric anticoagulation for suspected pulmonary embolism until a CT scan ruled it out. After a trial of diuretics without improvement in oxygenation, the patient was intubated and nitric oxide was initiated to facilitate transfer to a tertiary care hospital.

**Figure 1:**
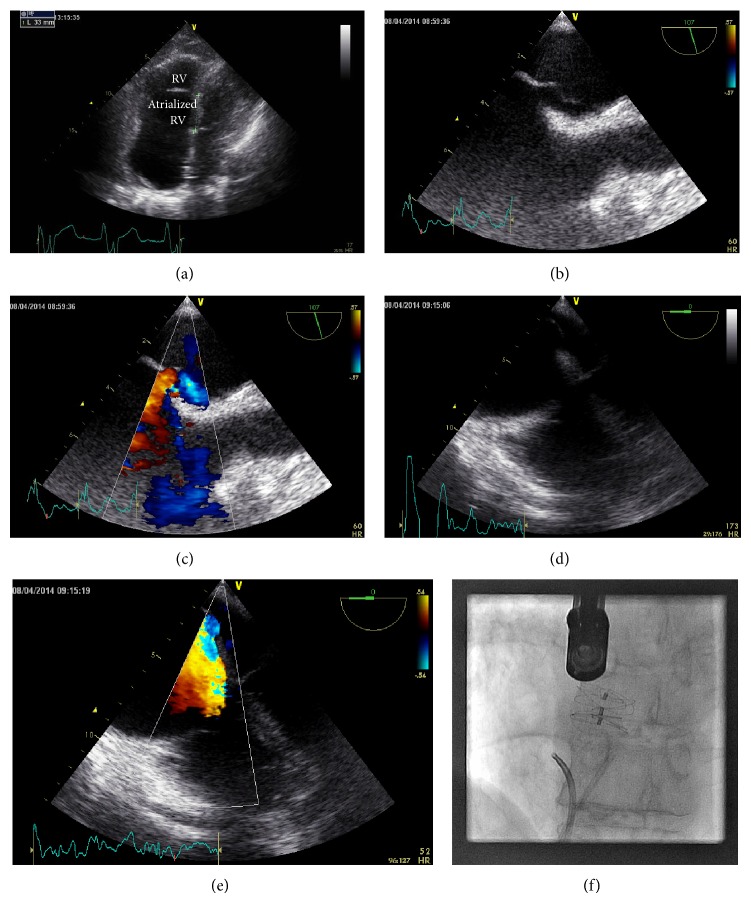
(a) TTE apical four-chamber view, focused on the right heart, showing Ebstein anomaly with atrialization of the right ventricle. (b) TEE image (107 degrees) showing the patent foramen ovale (arrow). (c) TEE image (107 degrees) with color Doppler, showing the patent foramen ovale. (d) TEE four-chamber view (0 degrees) showing the tricuspid valve and the patent foramen ovale. (e) TEE four-chamber view (0 degrees) with color Doppler, showing the eccentric tricuspid regurgitation jet flowing through the patent foramen ovale. (f) Coronary angiogram showing a 20 mm Gore Septal Occluder device used for partial percutaneous patent foramen ovate closure.

Postintubation transthoracic echocardiography revealed normal left ventricular systolic function, severely enlarged right ventricle with apical displacement of the tricuspid valve leaflets (consistent with Ebstein anomaly) (Figure 1(a)), severe tricuspid regurgitation, and a patent foramen ovale with right-to-left shunt at rest. Examination revealed an oxygen saturation of 80% on 100% fraction of inspired oxygen and high dose nitric oxide with evidence of peripheral cyanosis.

The patient underwent right heart catheterization demonstrating marginally elevated pulmonary artery pressure (mean pulmonary artery pressure 23 mmHg), elevated right atrial pressure (mean right atrial pressure 12 mmHg), and an oxygen step-down from the pulmonary veins to left ventricle. Transesophageal echocardiography confirmed significant flow of the tricuspid regurgitation jet through the patent foramen ovale (Figures 1(b) to 1(e), video).

Percutaneous closure of the patent foramen ovale was undertaken with a 30 mm Gore Septal Occluder device. The defect was not measured with a sizing balloon and the 30 mm device was selected to ensure complete defect coverage. This led to resolution of shunting and instantaneously improved arterial saturations (Table 1); however, the patient had significant deterioration in overall hemodynamics (20 mmHg systolic drop in systemic pressure, central venous pressure increase from 11 to 17, and mixed venous oxygen saturation of 35%). A 10 mm smaller device (20 mm Gore Septal Occluder) was selected for partial closure of the patent foramen ovale resulting in acceptable hemodynamics and reduction in right-to-left shunting (Figure 1(f)). The patient recovered, had no evidence of worsening right heart failure or peripheral edema, and was discharged home. Transthoracic echocardiography performed 3 months after patent foramen ovale closure demonstrated a moderate residual shunt present with right-to-left passage of saline contrast at rest.

## 3. Discussion

Partial atrial septal defect repair using fenestrations has been a technique used in neonates and infants with Ebstein anomaly; however, it has yet to be described in the adult population [3]. This method of repair is to prevent right ventricular collapse in neonates with high pulmonary vascular resistance and presence of small, poor functioning right ventricle. Additionally, performing a partial atrial septal defect repair ensures maintenance of cardiac output [3, 4].

In our case, it was thought that the initial transthoracic echocardiography 2 years prior to admission likely underestimated the eccentric tricuspid regurgitation jet. The transthoracic echocardiography and right ventriculography done on admission showed a small, hypercontractile right ventricle. The acute decompensation at the time of intubation was secondary to right-to-left shunting. She had no signs of chronic cyanosis (secondary erythrocytosis or clubbing). The decision to close the patent foramen ovale percutaneously was undertaken as the patient was at high risk for open heart surgery with tricuspid valve repair/replacement.

This case demonstrates that, in some adult patients with Ebstein anomaly and longstanding atrial septal defect/patent foramen ovale, shunt dependency may develop, as described in the pediatric population. In cases of persistent hypoxemia, they may benefit from partial atrial septal defect/patent foramen ovale closure. Further studies are needed to determine long-term efficacy.

## 4. Conclusion

In conclusion, we describe a case of Ebstein anomaly presenting with right-to-left shunting due to an eccentric tricuspid regurgitation jet, leading to hypoxemia. Complete patent foramen ovale closure led to acute decompensation; however, partial closure led to hemodynamic stability and improved oxygenation. We feel that similar patients with “patent foramen ovale dependency” from longstanding shunts may benefit from partial patent foramen ovale closure.

## Supplementary Material

Transesophageal echocardiogram demonstrating the severe tricuspid regurgitation with preferential shunting through the patent foramen ovale.

## Figures and Tables

**Table 1:** 

PFO closure hemodynamics			
	Predevice (FiO_2_ 0.8)	30 mm GSO device (FiO_2_ 0.8)	20 mm GSO device (FiO_2_ 0.4)
LV saturation	77.6%	96%	95.6%
Mixed venous saturation	—	37.5%	50%
RA pressure (a/v, mean), mmHg	9/13 (7)	—	17/25 (14)
PA pressure (mean), mmHg	—	22/11 (15)	35/14 (22)
